# Supplementary material for: A New BODIPY-Based Receptor for the Fluorescent Sensing of Catecholamines
Source: Molecules. 2024 Aug 5;29(15):3714. doi: 10.3390/molecules29153714 (PMC11314322; doi:10.3390/molecules29153714)
Supplement: Supplementary file 1 [file molecules-29-03714-s001.zip › molecules-3124619-supplementary.pdf]

New Bodipy-based receptor for the fluorescent sensing of catecholamines

Roberta Puglisi, Alessia Cavallaro, Andrea Pappalardo, Manuel Petroselli, Rossella Santonocito and Giuseppe Trusso Sfrazzetto

Table of contents

^{1}H-NMR, ^{13}C-NMR and ESI-MS of **(2)**	S2
UV-Vis Characterization of (**2**)	S3
Procedure for fluorescence titrations	S4
Determination of Stoichiometry	S4
Fluorescence titration between **(2)** and DA	S4
Job's Plot between **(2)** and DA	S5
HypSpec Plot of **(2)** and DA	S5
Fluorescence titration between **(2)** and NE	S4
Job's Plot between **(2)** and NE	S4
HypSpec Plot of (2) and NE	S4
DFT calculation	S6
Procedure for sensing by strip test	S8
Recovery	S8

Receptors **(1)** was synthesized as previous reported.[1]

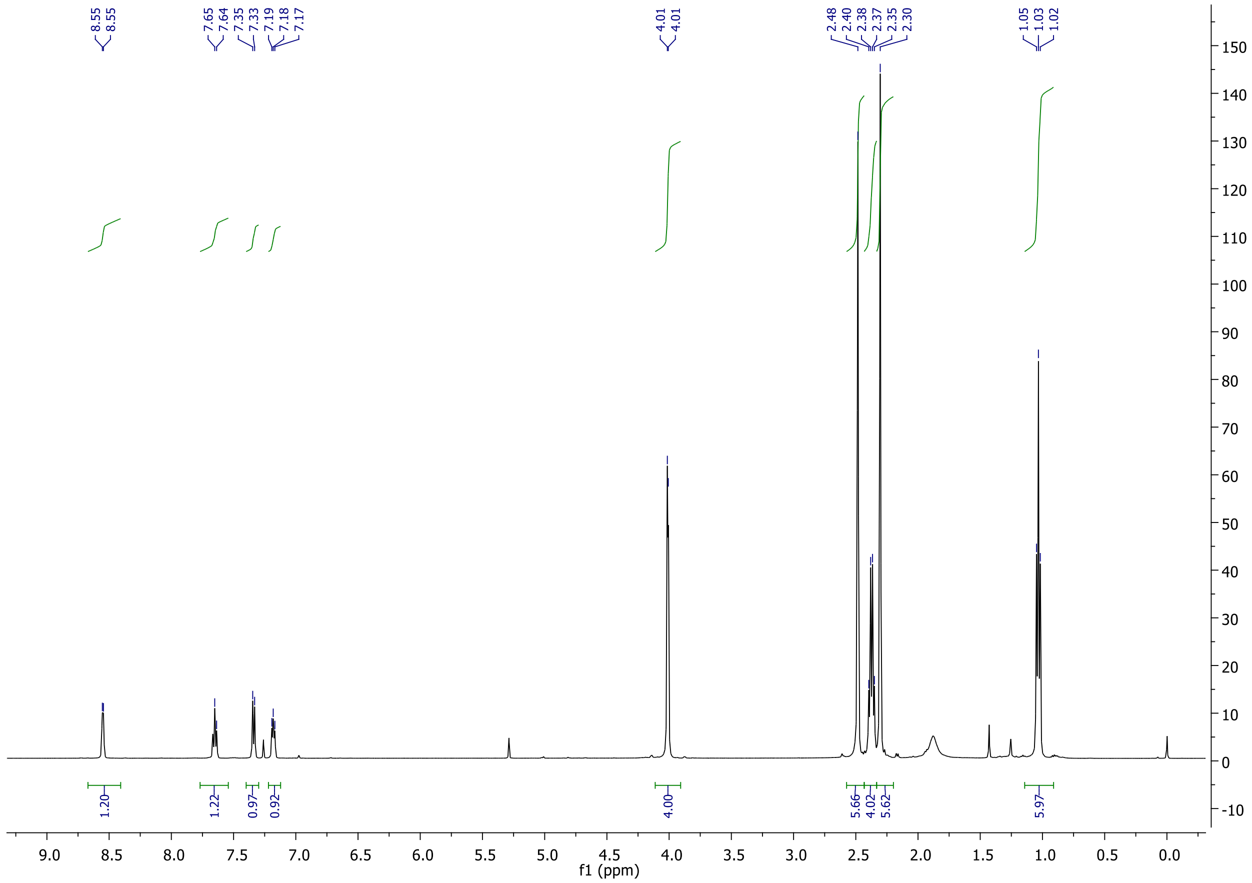

Figure S1. ^{1}H-NMR of (**2**) in $CDCl_3$.

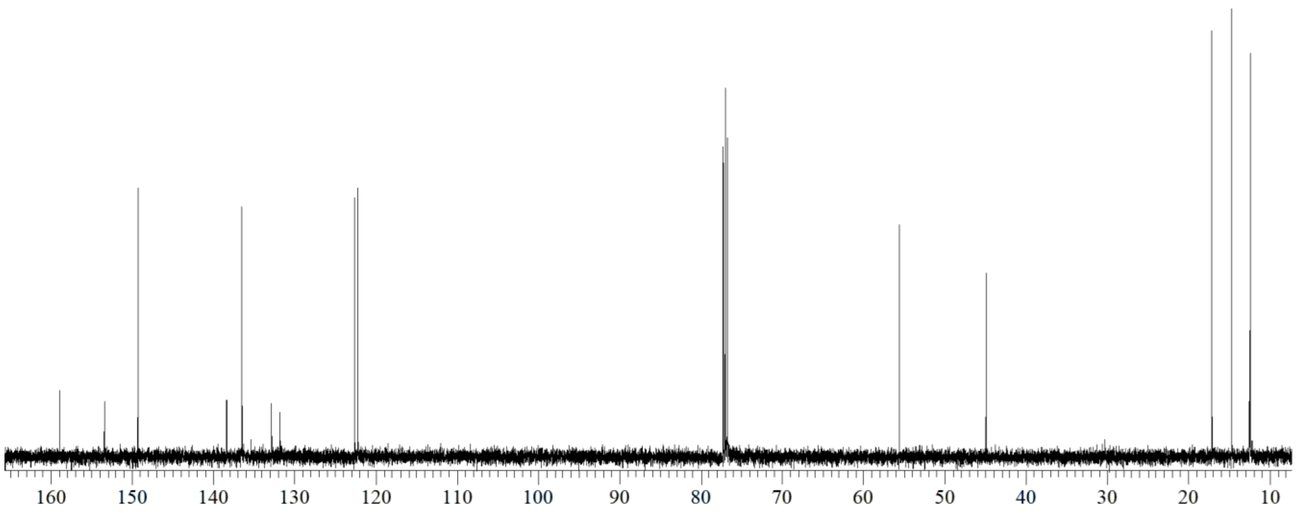

Figure S2. ^{13}C-NMR of bodipy (**2**) in $CDCl_3$

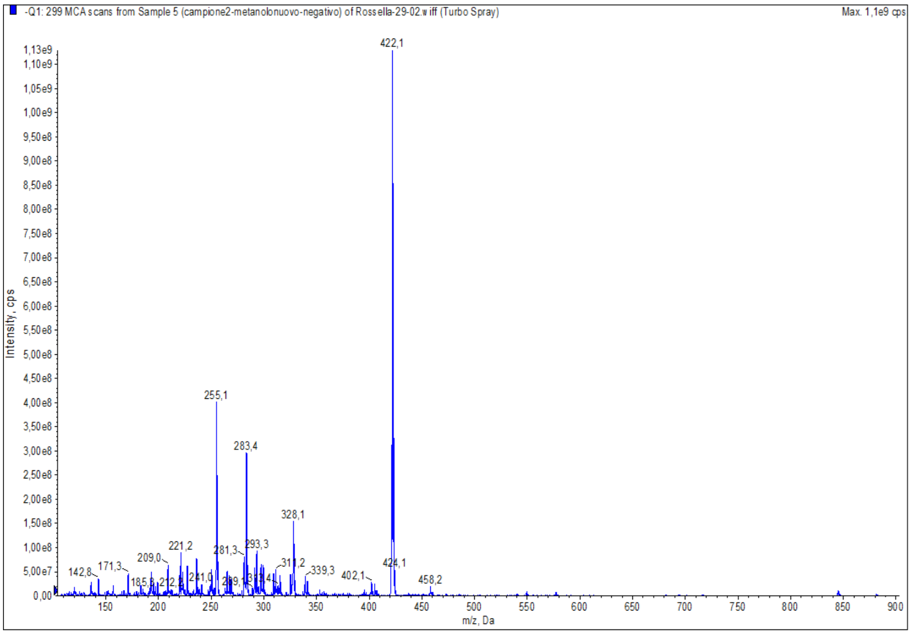

Figure S3. ESI-MS of (**2**) in CH_3CN.

UV-Vis characterization

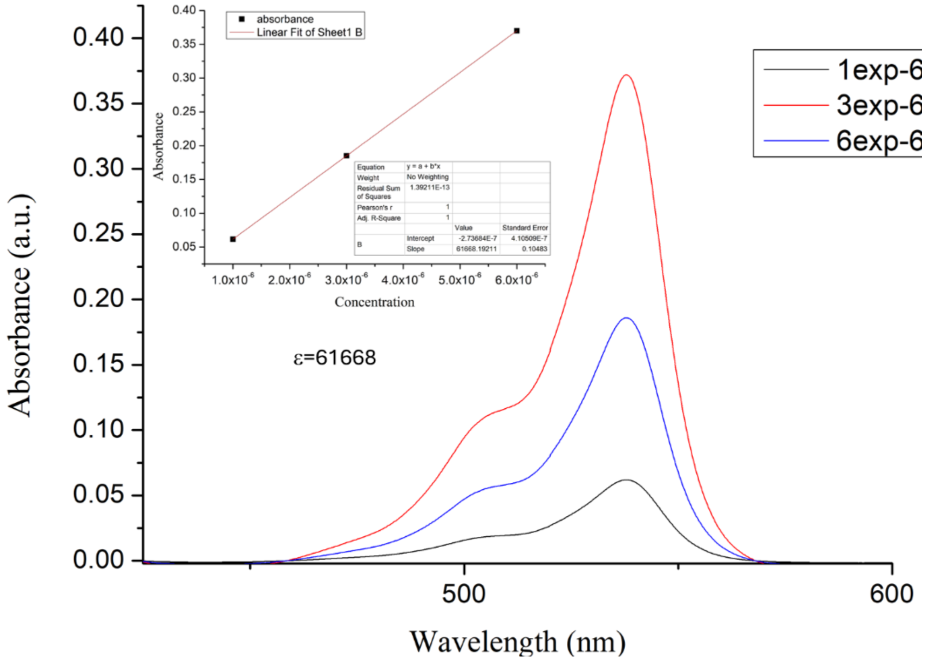

Figure S4. UV-Vis spectra of (**2**) at different concentration in $CHCl_3$

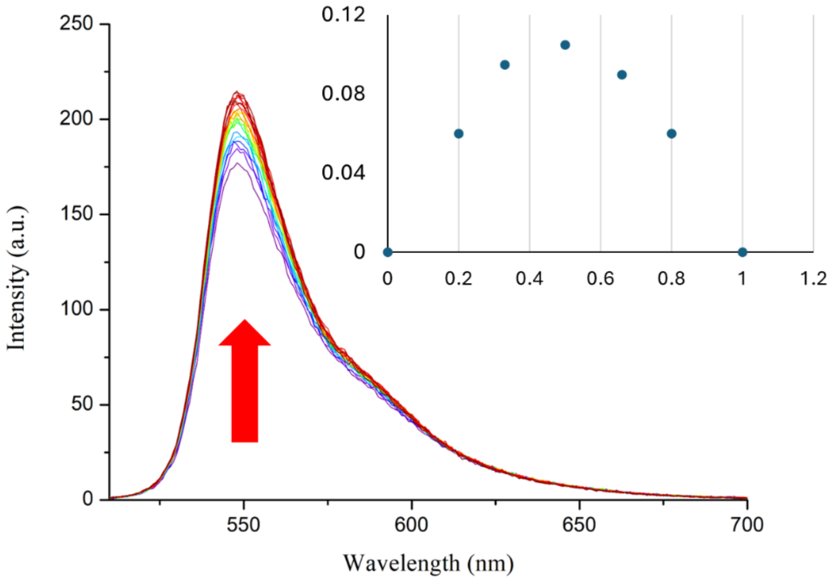

Figure S5. Fluorescence titration between (2) and DA ($CHCl_3$, [2] = 1 x 10^{-5} M, DA additions were in the 0-12 equivalent range). Inset shows the relative Job's Plot.

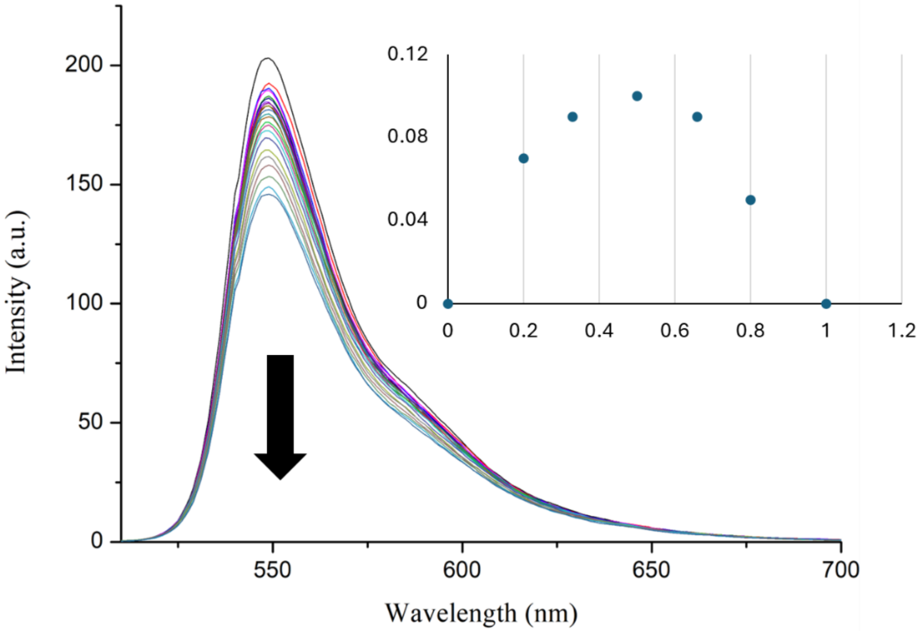

Figure S6. Fluorescence titration between (2) and NE ($CHCl_3$, [2] = 1 x 10^{-5} M, NE additions were in the 0-12 equivalent range). Inset shows the relative Job's Plot.

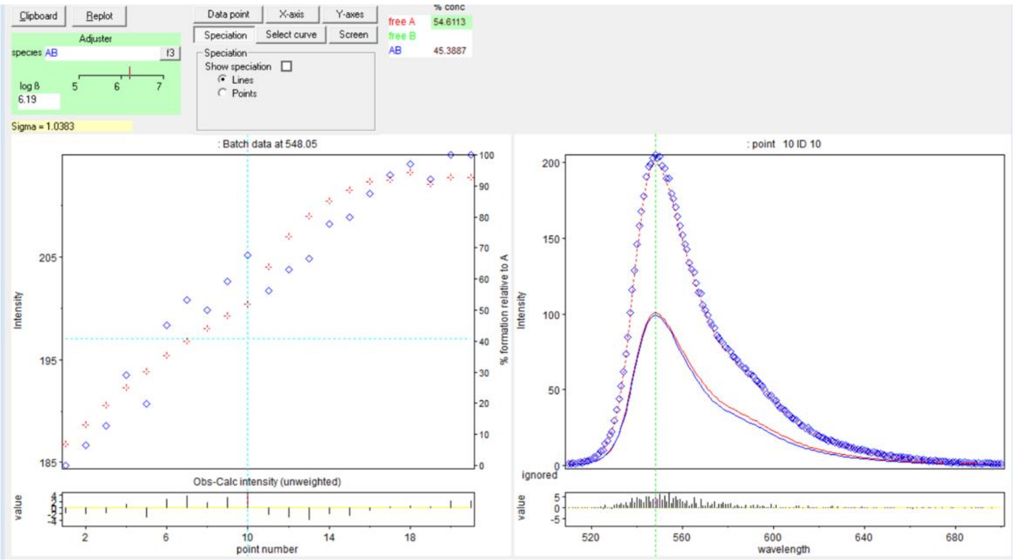

Figure S7. HypSpec Plot and relative output file

Converged in 1 iterations with sigma = 1.0385

standard

Log beta value deviation

AB 6.1901 0.0139

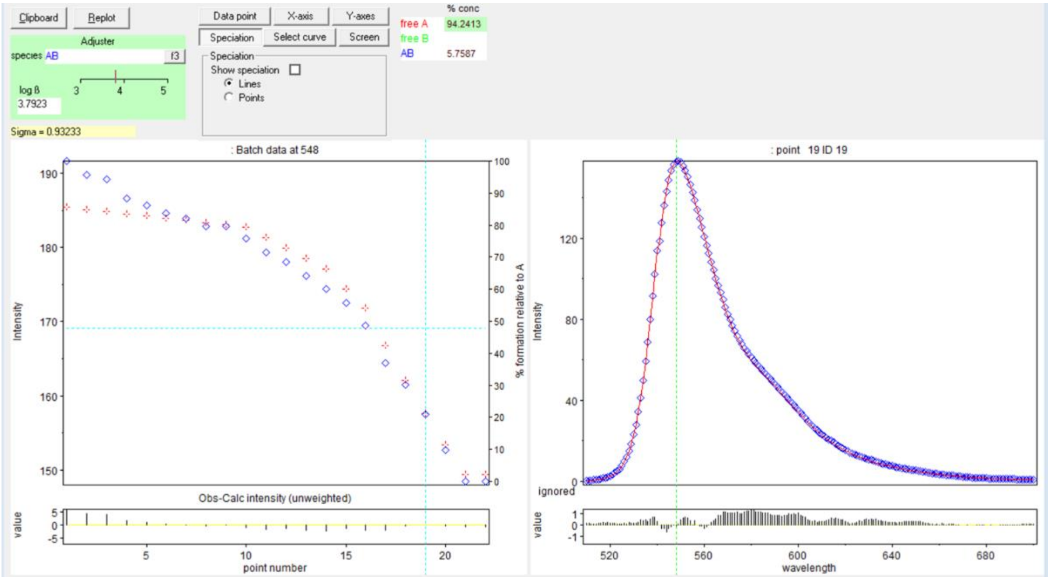

Figure S8. HypSpec Plot and relative output file

Converged in 1 iterations with sigma = 0.93244

standard

Log beta value deviation

AB 3.7923 0.0042

DFT Calculations

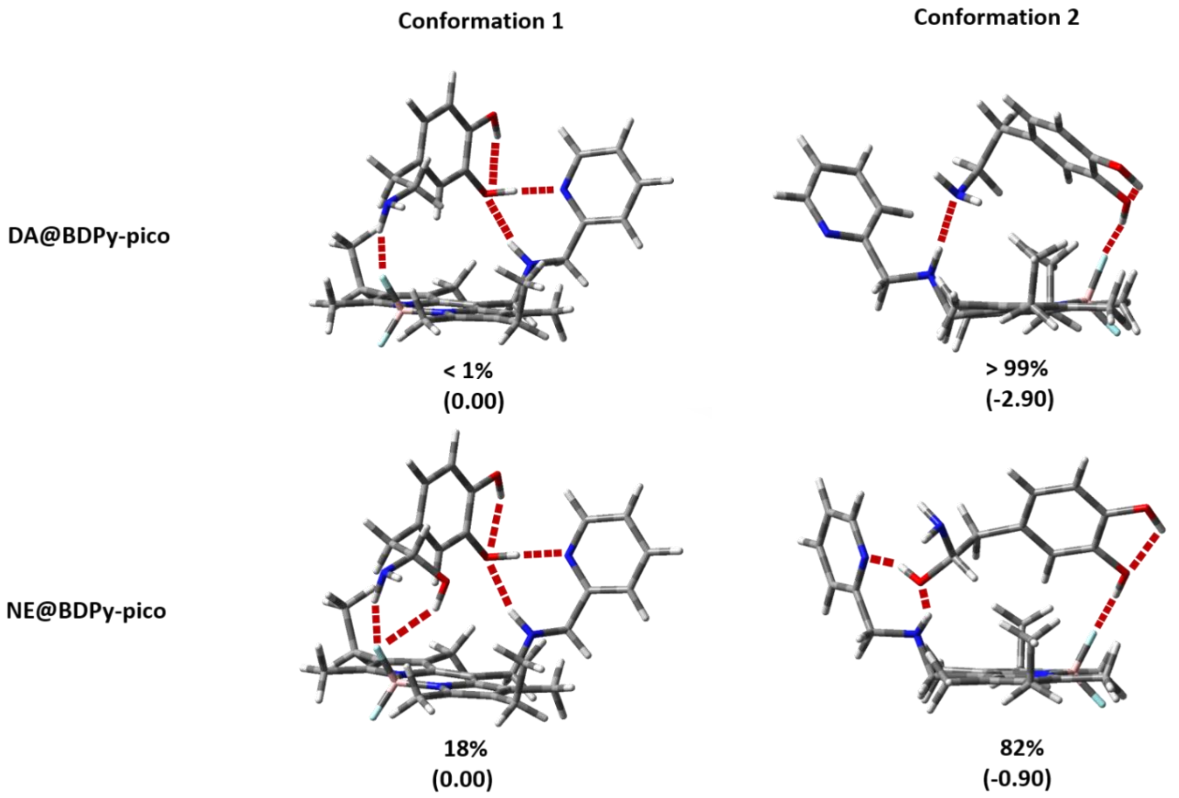

Figure S9. Most stable conformers for DA@2 (top) and NE@2 (bottom) calculated at the B3LYP/6-31G(d,p) level of theory in gas pahse. Hydrogen bonds in each conformer are marked in red. Boltzmann distribution is reported below each structure while relative energy (ΔE) is reported between parentheses, taking into account the conformation 1 as reference.

Table S1. Complexation energy ($E_{complex}$) for the DA@2 and NE@2 complexes in the two main conformation, showed in Figure SX1, calculated at the B3LYP/6-31g(d,p) level of theory in gas phase.

**Complex**	**$E_{complex}$ (kcal/mol)**	
	**Conformation 1**	**Conformation 2**
**DA@2**	33.8	36.7
**NE@2**	20.4	21.3

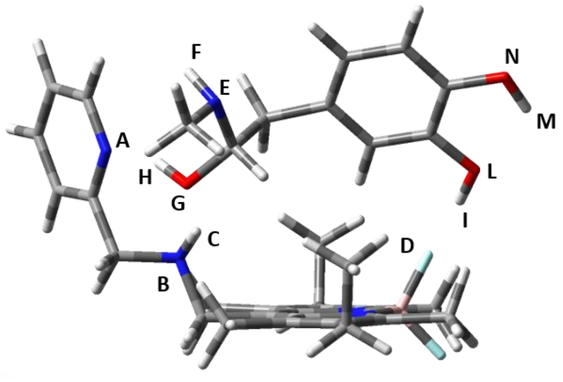

Entry	Atoms	DA@2	NE@2
1	A	-0,483	-0,521
2	B	-0,562	-0,528
3	C	0,314	0,279
4	D	-0,342	-0,346
5	E	-0,627	-0,571
6	F	0,252*	0,232*
7	G	-	-0,577
8	H	-	0,324
9	I	0,347	0,343
10	L	-0,629	-0,632
11	M	0,335	0,332
12	N	-0,578	-0,576

Figure S10. Optimized geometry of the model HG complex (adrenaline@**2**) and the relative Mulliken partial charges for each hydrogen bond donor and acceptor (A-N), calculated at the B3LYP/6-31G(d,p) level of theory in gas phase. Adrenaline has been taken into account as model HG compound for better clarity and discriminate the hydrogens in the terminal NH_2 group of **DA** and **NE** (atom F).
* Averaged value from NH_2 group.

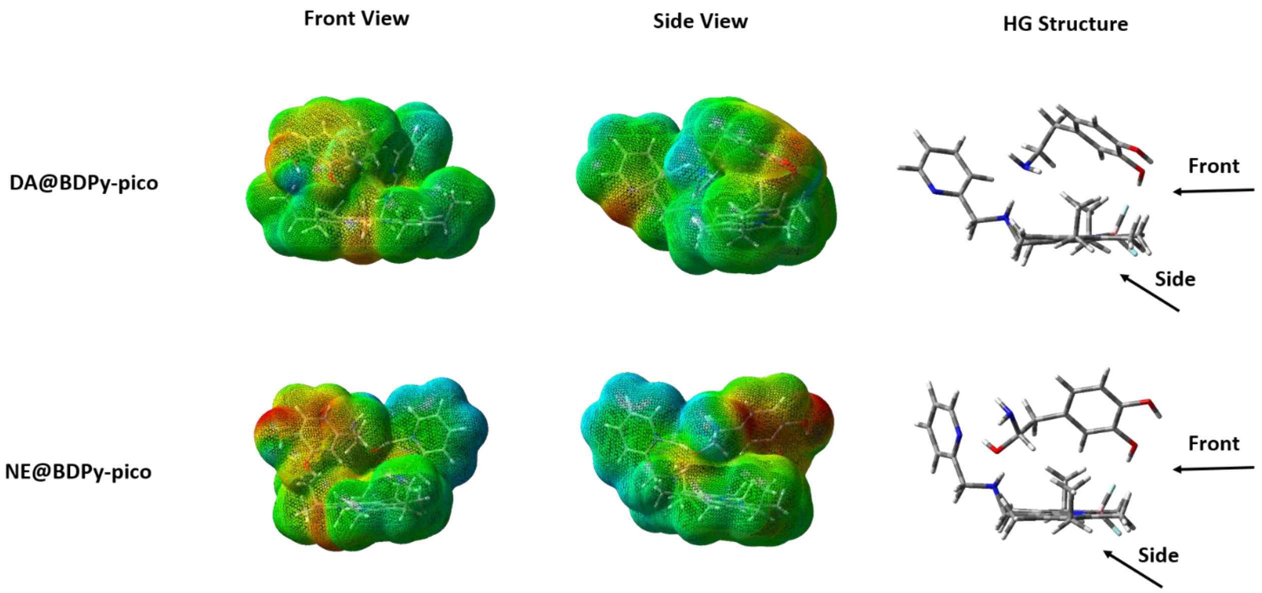

Figure S11. Electrostatic potential surface (ESP) for **DA@2** (top) and **NE@2** (bottom), calculated at the B3LYP/6-31G(d,p) level of theory in gas phase. Structures of the involved HG complexes are reported on the right, highlighting the prospective used in the ESP maps.

Procedure for sensing by strip test.

Three different solutions at different concentrations (from 10 μM to 1 NM) of DA and NE were prepared in MilliQ water. Three strip sensors were exposed to each solution and the emission spectra before and after the exposure of the analyte were acquired as reported in. A blank was also registered, consisting of MilliQ water (I_{water}). For statistical treatment, the following formula was applied: $(I_{water} - I_{analyte})/I_{BDPy}$, where $I_{analyte}$ is the emission of (2) probe after the exposure to analyte, I_{Water} is the emission of (2) probe after the exposure to MilliQ water and I_{BDPy} is the emission of (2) probe before the exposure to any analyte.

Optical fibre as detector.

We use an optical fibre as a detector, as a spectroscopic measurement system can be divided into the following components: a light source (I), a sample holder (II), a detection unit (III), and a control unit (IV). In particular: these are the optical components used for analysis with the optical fibre as the detector from Thorlabs.

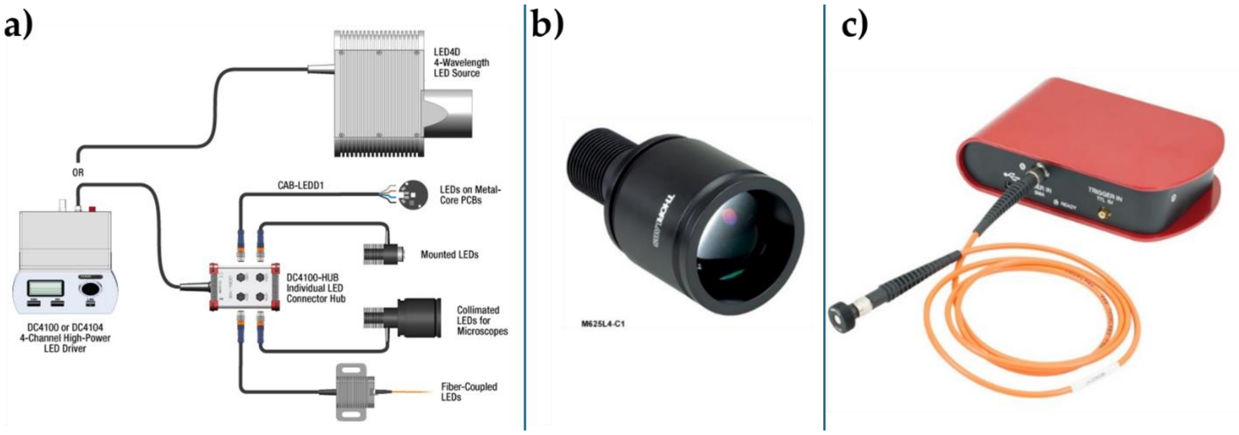

Figure S12. a) Advanced Four-Channel LED Driver. b) LED: 365 nm, 430 mW (Typ.) Collimated LED for Zeiss Axioskop & Examiner, 1000 mA. c) Compact CCD Spectrometer, 350 - 700 nm, Metric

Recovery. The recovery of the strip test was tested by performing an analyte-water cycles. Firstly, DA (1 μM in water) was dropped on the probe (2), which was dried in air for 30 seconds and emission intensity have been acquired as reported above. Then the probe of the strip test was exposed to water for 1 minute. At the end, the emission intensity has been acquired. The cycle was repeated five times, and the same thing was done for the NE.

The Figure S15 shows an example of emission spectra collected by an optical fibre. In particular, the emission spectrum in blue represents the emission of the probe before exposure of the analyte, the emission spectrum in red represents the emission of the probe after exposure of the analyte, and the emission spectrum in black represents the emission spectrum of the probe after exposure of water. As we can see, the probe (2) after water exposure restores its initial emission.

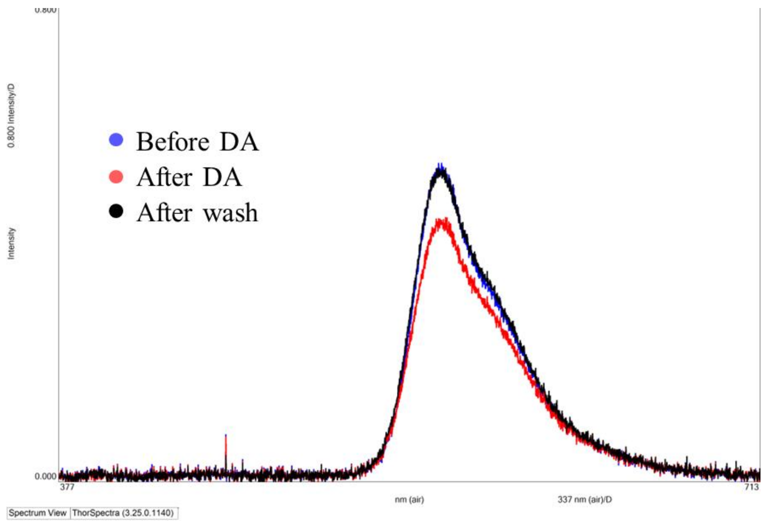

Figure S13. Emission spectra collected by an optical fiber

[1] Santonocito, R.; Spina, M.; Puglisi, R.; Pappalardo, A.; Tuccitto, N.; Trusso Sfrazzetto, G.; Chemosensors, 2023, 11, 503.
